# How social media expression can reveal personality

**DOI:** 10.3389/fpsyt.2023.1052844

**Published:** 2023-03-02

**Authors:** Nuo Han, Sijia Li, Feng Huang, Yeye Wen, Yue Su, Linyan Li, Xiaoqian Liu, Tingshao Zhu

**Affiliations:** ^1^Chinese Academy Sciences Key Laboratory of Behavioral Science, Institute of Psychology, Chinese Academy of Sciences, Beijing, China; ^2^Department of Psychology, University of Chinese Academy of Sciences, Beijing, China; ^3^School of Data Science, City University of Hong Kong, Hong Kong, Hong Kong SAR, China; ^4^Department of Social Work and Social Administration, The University of Hong Kong, Hong Kong, Hong Kong SAR, China; ^5^School of Electronic, Electrical, and Communication Engineering, University of Chinese Academy of Sciences, Beijing, China; ^6^Department of Infectious Diseases and Public Health, Jockey Club College of Veterinary Medicine and Life Sciences, City University of Hong Kong, Hong Kong, Hong Kong SAR, China

**Keywords:** personality, social media, machine learning, domain knowledge, psychological lexicons, mental health, Big Five

## Abstract

**Background:**

Personality psychology studies personality and its variation among individuals and is an essential branch of psychology. In recent years, machine learning research related to personality assessment has started to focus on the online environment and showed outstanding performance in personality assessment. However, the aspects of the personality of these prediction models measure remain unclear because few studies focus on the interpretability of personality prediction models. The objective of this study is to develop and validate a machine learning model with domain knowledge introduced to enhance accuracy and improve interpretability.

**Methods:**

Study participants were recruited *via* an online experiment platform. After excluding unqualified participants and downloading the Weibo posts of eligible participants, we used six psycholinguistic and mental health-related lexicons to extract textual features. Then the predictive personality model was developed using the multi-objective extra trees method based on 3,411 pairs of social media expression and personality trait scores. Subsequently, the prediction model’s validity and reliability were evaluated, and each lexicon’s feature importance was calculated. Finally, the interpretability of the machine learning model was discussed.

**Results:**

The features from Culture Value Dictionary were found to be the most important predictors. The fivefold cross-validation results regarding the prediction model for personality traits ranged between 0.44 and 0.48 (*p* < 0.001). The correlation coefficients of five personality traits between the two “split-half” datasets data ranged from 0.84 to 0.88 (*p* < 0.001). Moreover, the model performed well in terms of contractual validity.

**Conclusion:**

By introducing domain knowledge to the development of a machine learning model, this study not only ensures the reliability and validity of the prediction model but also improves the interpretability of the machine learning method. The study helps explain aspects of personality measured by such prediction models and finds a link between personality and mental health. Our research also has positive implications regarding the combination of machine learning approaches and domain knowledge in the field of psychiatry and its applications to mental health.

## 1. Introduction

Personality uniquely characterizes an individual. According to the psychological definition of this term, personality refers to an individual’s particular combination of emotional, attitudinal, and behavioral response patterns (1). Broadly, personality can influence a wide range of types of human activity and behavior, such as mental states and social behavior (2, 3). Besides, personality has been found could potentially be related to psychiatric disorders, and dimensional personality models have implications for psychiatric diagnosis and treatment around the world (4). Therefore, the study of personality is of great importance in the field of psychology and psychiatry. As stated by WHO, there is an extreme resource shortage regarding diagnosing and treating people with mental health problems (5). Personality, as a potential predictor of mental health-related outcomes (6), is urged to be given more attention in the context of providing timely and effective targeted interventions.

A traditional strategy for measuring personality requires participants to answer a series of questions (typically ranging from 20 to 360) that evaluate their behavior and preferences (7, 8). This self-reported method has a solid theoretical foundation and is a well-known measurement of personality in psychology. However, self-reporting is not the best choice in some specific scenarios (such as those needs that require large-scale measurement or the real-time acquisition of personality traits). In addition, although the “L-scale” is rigorously designed in personality surveys, our ability to accurately measure people’s true personality scores remains limited, which is unavoidable given the complexity of humanity (9, 10).

To address these issues, much machine learning research on personality assessment has focused on the online environment (11–15). The exponential increase in the amount of data people generate online has allowed researchers to unobtrusively gather and automatically predict the personality traits of social media users. Predicting personality traits using social media may represent a rapid, cost-effective alternative to surveys and may allow us to reach larger populations (6). Moreover, the individual’s level of social media use is often discretionary rather than mandated and is thus more likely to reflect personal motives, needs, values, preferences, and other personality attributes (16), which offers a better picture of people’s personality traits in their daily lives.

However, although previous research has confirmed that machine learning approaches offer an unprecedented opportunity to advance personality assessment, the aspects of personality these prediction models measure remain unclear (14, 17). This issue also corresponds to a concern in the field of computer science; that, is, ways of improving the interpretability of prediction models (18). In other words, although the accuracy of the personality prediction model has been repeatedly broken through, the interpretability of the model is still a problem that needs attention. This study targets this previously unexplored topic in an attempt to introduce domain knowledge to improve the interpretability of the prediction model while ensuring prediction accuracy.

We conducted our study on Sina Weibo, a leading Chinese social media platform featuring more than 926 million registered users (19). After conducting the literature review, we finally chose six psycholinguistic, psychological, and mental health-related lexicons to extract linguistic features (the detailed reasons for lexicon selection are listed in the section “2. Materials and methods”). Subsequently, we used multi-objective learning to identify the empirical associations between linguistic features and personality traits within specific samples. The prediction model was ultimately validated using reliability and validity tests for the psychological scales used. Our study has positive implications regarding using social media to predict personality traits in non-professional scenarios such as online large-scales. It can also help us understand the mental health and high-risk factors associated with internet users. Furthermore, our research has a positive effect on the combination of machine learning approaches and domain knowledge in the field of psychiatry.

## 2. Materials and methods

### 2.1. Participants

This study used a self-developed online experimental platform to recruit participants. Between May 2011 and October 2014, a total of 3,886 Weibo users participated in the experiment. To ensure that the participants were active users of Weibo and that the psychological questionnaire was valid, this study excluded (1) participants who had posted fewer than 500 Weibo posts since their accounts were created and (2) participants whose completion time for the questionnaire was too long or too short (e.g., participants who completed the questionnaire is less than 30 s). Finally, 3,411 samples were included in this study, including 1,278 males, 2,059 females, and 74 participants who preferred to not report their gender.

### 2.2. Instruments

#### 2.2.1. Online experimental platform

**FIGURE 1:**
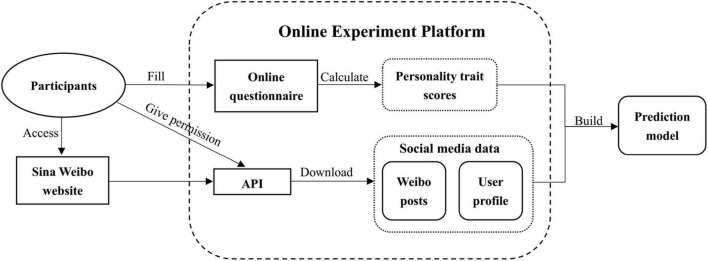
Information collection process using the online experiment platform.

As mentioned previously, we used an online experimental platform to retrieve social media data from the participants as discussed above, including information from their user profiles, their posts, and their online questionnaire results. The platform is a Web Access Connection to Sina Microblog, in which context participants were able to log on to the platform using their Sina Microblog account. The information collection process using this online experiment platform is shown in Figure 1. The privacy of users was strictly ensured throughout this process in accordance with the ethical principles suggested by Kosinski et al. (20). The ethical conduct of the research was approved by the Institutional Review Board at the Institute of Psychology, Chinese Academy of Sciences, under code H15009.

#### 2.2.2. The Big Five inventory

Among the conceptual frameworks used for personality research, the Big Five model is generally regarded as unified and parsimonious and is the most commonly used model in this context (21, 22). Empirical studies have verified the overall factor structure and integrity of the Big Five constructs of Openness (Open.), Conscientiousness (Cons.), Extraversion (Extr.), Agreeableness (Agr.), and Neuroticism (Neu.) in many different settings and fields of inquiry (7, 23). Explanations of each trait are summarized in Table 1.

**TABLE 1:** Explanation of the Big Five personality traits.

Trait	Description	Item examples
Openness	Openness describes the breadth, depth, and complexity of an individual’s spiritual and life experience as well as his or her degree of tolerance for or acceptance of new things and ideas. It includes characteristics such as fantasy, art, feeling, innovation, interest, and value.	Q5. Is original, comes up with new ideas. Q10. Is curious about many different things.
Conscientiousness	Conscientiousness describes the individual’s adherence to tasks and impulse control over non-goal behaviors and is a way of controlling, managing, and regulating the individual’s impulses. It includes characteristics such as competition, order, achievement, obligation, self-discipline, and meticulousness.	Q3. Does a thorough job. Q8. Can be somewhat careless.
Extraversion	Extraversion describes the individual’s level of involvement and activity with respect to interpersonal interactions, dominance, sociality, expressiveness, and positive emotions. It includes traits such as being enthusiastic, generous, confident, and active.	Q1. Is talkative. Q6. Is reserved.
Agreeableness	Agreeableness describes a soft attitude toward others, empathy and concern for others, and a tendency to desire approval from others. It includes traits such as altruism, gentleness, trust, honesty, compliance, and humility.	Q17. Has a forgiving nature. Q22. Is generally trusting.
Neuroticism	Neuroticism describes an individual’s perception of negative emotions, the ability to regulate such emotions, and level of emotional stability. It includes negative emotional traits such as anxiety, anger, depression, selfishness, impulsiveness, and vulnerability.	Q14. Can be tense. Q24. Is emotionally stable, not easily upset.

**FIGURE 2:**
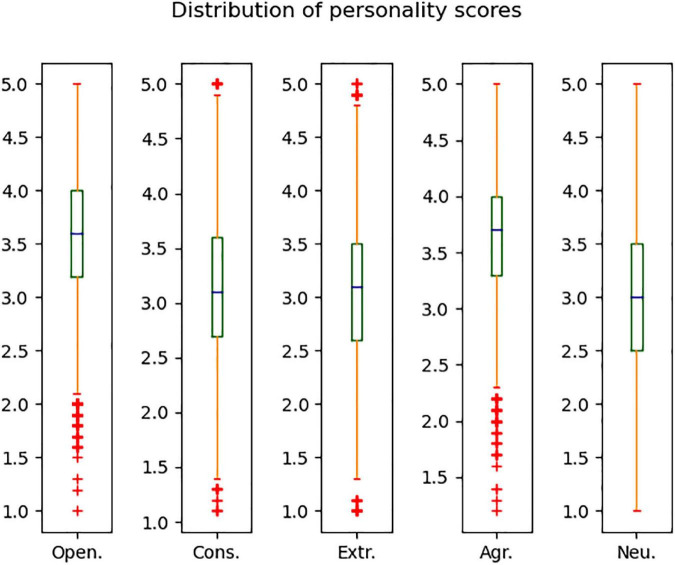
The distribution of participants’ personality trait scores.

The BFI-44 is one of the most generally used brief measures of the Big Five personality traits (24). This research used the Chinese version of John O.’s 44-item Big Five Inventory (BFI-44).^1^ It contains five subsets, which measure Open. with 10 items, Cons. with 9 items, Extr. with 8 items, Agr. with 9 items, and Neu. with 8 items. Participants answer each question on a 5-point Likert scale, ranging from Disagree strongly (1 point) to Agree strongly (5 points). The reliability of BFI-44 has been tested by previous studies, and the α coefficients of each dimension are close to 0.8 (24). When the participant finishes the scale, the scores of the five subsets can be obtained. Thereafter, the five dimensions of participants’ personalities can be represented using the average score of each subset. The boxplot in Figure 2 shows the distribution of participants’ personality scores for each trait.

#### 2.2.3. Linguistic lexicons

This study targets using machine learning methods to identify interpretability issues related to personality, and with the expectation that the model would be beneficial for mental health diagnosis, we chose to use a series of psychological and mental health-related lexicons to extract features that might be related to personality. Specifically, we used the Simplified Chinese version of the Linguistic Inquiry and Word Count (SC-LIWC) (25), the Weibo Basic Mood Lexicon (Weibo-5BML) (26), the Chinese Suicide Dictionary (CSD) (27), the Moral Motivation Dictionary (MMD) (28), the Moral Foundations Dictionary (MFD) (29), and the Culture Value Dictionary (CVD) (30). We introduce these lexicons and explain why we chose them as follows.

##### 2.2.3.1. SC-LIWC

Linguistic Inquiry and Word Count is widely used in natural language processing (NLP) to map the psychological and linguistic dimensions of linguistic expressions. In this research, we used SC-LIWC, which reports 87 dimensions of language use in simplified Chinese (25). The validity of SC-LIWC has been validated with respect to the detection of psychological expressions in short texts on social media (31). In fact, the use of LIWC to extract language features is a longstanding practice among scientists. Scientists have used LIWC or SC-LIWC to construct computational prediction models of psychological traits, including personalities (12, 32), mental health status (33, 34), and subjective wellbeing (35, 36).

##### 2.2.3.2. Weibo-5BML

Weibo-5BML contains 818 Chinese words (phrases) that can be annotated with 5 emotions (happiness, sadness, anger, fear, and disgust) (26). Previous works have used this lexicon to identify mood changes in Weibo users and verified its reliability (37). The underlying rationale for including emotion-related features is that people with different personality traits tend to express themselves differently and hence use different words (phrases) and express different emotions. A relationship between emotions and personality traits has also been observed in previous research (38). Some studies have also used emotional lexicons to facilitate the task of personality prediction (39, 40).

##### 2.2.3.3. CSD

Chinese Suicide Dictionary is intended to identify suicide risks on social media. It can be used to collect suicidal expressions from social media posts. CSD is composed of 2,168 words, which can be classified into 13 different categories (e.g., “hostility words,” “self-regulation words,” and “personality words”) (27). Li et al. (41) used CSD to measure users’ risk of suicidal ideation on Weibo, proving that CSD is reliable. We selected this lexicon because academic works have found that probable or definite personality disorders are related to a positive attitude toward suicide (42).

##### 2.2.3.4. MMD

In this study, we used the simplified Chinese version of the MMD that was developed by Zhang and Yu (28). It includes 690 agency words and 260 communion words. MMD has been used to measure the moral motivations of groups on social media in many psychological studies, such as that conducted by Zhao et al. (43). The reason we chose this dictionary is that previous studies have claimed to identify personality-level differences in morality and honesty (44–46).

##### 2.2.3.5. MFD

Moral Foundations Dictionary can reflect the extent to which people follow basic moral norms in terms of their language habits (29). This study used the simplified Chinese version of the MFD, which contains 590 Chinese words or phrases ranging across 6 dimensions (harm, fairness, ingroup, authority, purity, and general morality) (47). With the exception of general morality, the other five dimensions all contain positive and negative word lists (47). Therefore, the MFD contains a total of 11 categories. This dictionary has been used to measure the moral foundations of users in social media studies (41, 48). Previous studies have suggested that personality is positively associated with moral values (49, 50).

##### 2.2.3.6. CVD

Culture Value Dictionary consists of 53 individualistic Chinese words, 64 collectivistic Chinese words, and their synonyms (30). This dictionary has been used to measure the spectrum of individualism-collectivism in previous psychological studies, such as those conducted by Han et al. (51) and Huang et al. (52). One hypothesis concerning personality development has claimed that personality development should vary with the social environment surrounding each culture (53, 54). Therefore, we also extracted features expressing cultural value to develop the personality prediction model.

### 2.3. Procedure

#### 2.3.1. Data collection

First, we randomly sent invitations to approximately twenty thousand Weibo users. Subsequently, users who were willing to participate in our experiment were instructed to use our online experiment platform to provide informed consent and complete a psychological questionnaire so that we could access their personality trait scores. Finally, the social media data of these users (Weibo posts and user profiles) were downloaded *via* the Sina Weibo API as shown in Figure 1.

#### 2.3.2. Data preprocessing

**FIGURE 3:**
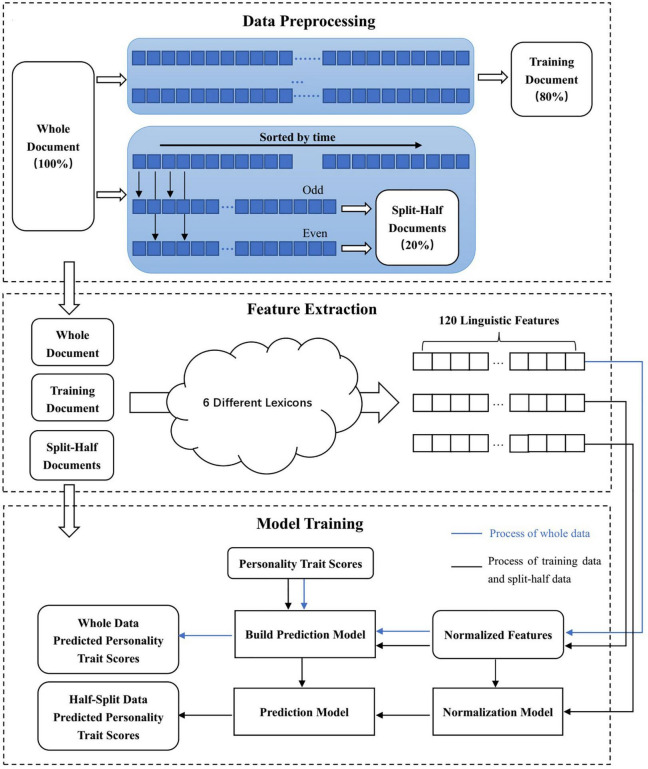
Data preprocessing, feature extraction, and model training.

Following data collection, the scores of the subscales in the BFI-44 were calculated. Subsequently, the total number of Weibo posts by all participants was counted, and only participants with more than 500 Weibo posts were retained for this study. The data preprocessing process is shown in Figure 3 for reference. We called the document containing the posts by these remaining participants the “whole” data. Thereafter, we randomly sampled 80% of the remaining participants. We merged all posts by the sampled participants into one document, which we called the “training” document. For the remaining 20% of participants, we sorted all posts by every participant in chronological order. Subsequently, we separately merged the sorted odd-numbered posts by each participant into one document and the sorted even-numbered posts by each participant into another document. We called the two documents containing the odd-numbered and even-numbered posts of this 20% of participants the “split-half” data.

#### 2.3.3. Feature extraction

As shown in Figure 3, for the “whole” data, “training” data, and “split-half” data, we extracted a wide variety of dictionary-based linguistic features from each document. As introduced in the “linguistic lexicons” section, these linguistic features included 87 LIWC features, 5 Weibo-5BML features, 13 CSD features, 2 MMD features, 11 MFD features, and 2 CVD features.

Referring to the calculation method for language features, we first combined all posts by each user into one pseudo long text and divided each pseudo long text into several word pieces. Subsequently, we calculated the frequency of word pieces from each lexicon category as language features. Equation 1 shows the specific word frequency calculation method, in which context *i* represents the i-the language feature and *j* represents the j-the user. *t*_*i,j*_ is the frequency with which the term in the i-the language category appears in the document, *w_j_* is the word count of the j-the user’s text, and *F*_*i,j*_ is the value of the i-the linguistic feature of the j-the user. Therefore, the larger the value of *F*_*i,j*_ is, the more frequently the i-the language feature occurs in the j-the user’s text.


(1) $$F_{i,j}=\frac{t_{i,j}}{w_{j}}$$

Following feature extraction, we created four feature files responding to four data documents. In every feature file, each row represented a participant, and each column represented a feature. Specifically, the “whole” feature file, the “training” feature file, and the two “split-half” feature files contained 3,411, 2,728, and 682 rows, respectively, all files contained 120 columns.

#### 2.3.4. Model training

During the first step of model training, all features were normalized to ensure that the contribution of features to models was not affected by their range and distribution. Since the sample size was much larger than the feature size, to retain as much feature information as possible, no feature selection or feature reduction was performed in this study.

Following feature normalization, we used multi-objective extra trees (MOET) to develop a regression prediction model. As an extension of the random forest regression model, the extra trees algorithm was proposed as a computationally efficient and highly randomized extension of the random forest algorithm (55). The extra trees algorithm is an important algorithm within the class of decision tree-based ensemble learning methods. It has been shown to exhibit state-of-the-art performance with respect to many regression tasks featuring high-dimensional inputs and outputs (56). This study used MOET because multi-objective learning could employ multiple object modeling strategies to improve performance beyond the level that could be achieved by single-object learning in the same context (32). During training, a MOET regression model featuring 1,000 trees which named the “full model” was trained using pairs of input linguistic features (120 dimensions) and annotated output personality trait scores. Fivefold cross-validation was used to adjust the model parameters. Besides, considering the ubiquitous use of the LIWC lexicon in personality prediction, we wanted to make sure if adding the other 5 lexicons worked. So we also built a regression model using only SC-LIWC features (87 dimensions) with the same training method and named it the “LIWC model.”

During the process of five-fold cross-validation, the test set’s predictive values for every fold were saved. Accordingly, all samples in the “whole” data were predicted once as test sets. Similarly, we first used the “training” data with all feature (120 dimensions) to develop a prediction model for 5 personality traits and then applied the prediction model to the odd-numbered and even-numbered posts “split-half” data. The overall process is shown in Figure 3. Ultimately, we obtained three sets of predicted values, i.e., 3,411 based on the “whole” data and 682 based on the “split-half” data.

### 2.4. Statistical analysis

We first conducted statistically analysis on the information drawn from user profiles. Second, considering the fact that a basic premise of the multi-objective approach is that these dimensions are correlated (even weakly) (57), we calculated the Pearson correlation coefficients among the five personality traits. Third, to ensure that the “full model” can perform better than the “LIWC model,” we compared the Pearson correlation coefficients between test scores and predicted scores of the two models, which were obtained from cross-validation. Furthermore, the researchers used the attributes of the extra trees algorithm to output the feature importance of the language features that were used for modeling. The importance of a feature indicates how important the feature was to the model-constructing process. The sum of the values of the importance of all features used is 1. The sum of the importance of each lexicon was calculated. Since total feature importance increases with the number of features, we also calculated the average feature importance for each lexicon, which is the ratio of the sum of the importance of each lexicon compared to the number of features included in each lexicon.

Finally, we structural validity and criterion validity based on the “whole” data results. The split-half reliability was obtained based on the “split-half” predicted scores. The method used in this step referred to the study conducted by Wang et al. (58). Specifically, multitrait-multimethod matrix analysis was conducted to explore the structural validity of the linguistic prediction model. Five personality traits were included in the multitrait-multimethod matrix, including openness, conscientiousness, extroversion, agreeableness, and neuroticism, and two methods were involved in this process, including the BFI-44 subscales and the linguistic prediction model. To conduct the analysis of criterion validity, the actual scores of each subscale were used as the effective standard. Subsequently, the Pearson correlation coefficients between the predicted values of the “whole” data and the actual scores of the corresponding subscales were calculated. Referring to the assessment of split-half reliability, the Pearson correlation coefficient between the predicted values of the two “split-half” data was calculated as an indicator of reliability. All the Pearson correlation coefficients calculated in the context of this study were determined using Statistical Product and Service Solutions (SPSS) 22.0 software (59).

## 3. Results

### 3.1. User profile information

User profile information was collected in this study. Among all participants, 60.3% were female. The follower counts with median counts of 515 (SD 3600.6), friend counts with median counts of 298 (SD 340), and post counts with median counts of 2630 (SD 2000.6) are displayed in Table 2.

**TABLE 2:** Basic profile information of participants.

Class	Type	Participants *n* (%)
Gender	Male	1,278 (37.5)
	Female	2,059 (60.3)
	Not report	74 (2.2)
Follower count	<100	155 (4.5)
	101–1,000	2,668 (78.2)
	1,001–10,000	487 (14.3)
	>10,000	27 (0.8)
	Missing data	74 (2.2)
Friend count	<100	250 (7.3)
	101–1,000	2,895 (84.9)
	>1,000	192 (5.6)
	Missing data	74 (2.2)
Post count	501–1,000	189 (5.5)
	1,001–10,000	3,195 (93.7)
	>10,000	27 (0.7)
Total		3,411 (100)

### 3.2. Correlations among personality traits

The Pearson correlation coefficient *r* describes the degree of linear correlation between two variables. The absolute value of *r* stands for the strength of the correlation. Table 4 shows that all the dimensions, with the exception of neuroticism, exhibited significant positive correlations with one another (see the blue background section). The neuroticism dimension was significantly negatively correlated with the other four dimensions.

**TABLE 3:** Pearson correlation results in cross-validation for each dimension.

Dimension	*R*_1_	*R*_2_
Open.	0.46***	0.39***
Cons.	0.48***	0.46***
Extr.	0.47***	0.40***
Agr.	0.44***	0.42***
Neu.	0.46***	0.43***

*R*_1_, the Pearson correlation coefficients between the predicted scores and the test score of the “full model” in cross-validation. *R*_2_, the Pearson correlation coefficients between the predicted scores of the “LIWC model” in cross-validation. *N* = 3,411.

***p < 0.001.

**TABLE 4:** Pearson correlations of each dimension.

		Prediction model					BFI-44				
		Open_1_	Cons_1_	Extr_1_	Agr_1_	Neu_1_	Open_2_	Cons_2_	Extr_2_	Agr_2_	Neu_2_
Prediction model	Open_1_	–									
	Cons_1_	0.31***	–								
	Extr_1_	0.09***	0.15***	–							
	Agr_1_	0.22***	0.47***	0.19***	–						
	Neu_1_	−0.30***	−0.60***	−0.39***	−0.59***	–					
BFI-44	Open_2_	0.46***	0.35***	0.10***	0.09***	−0.14***	–				
	Cons_2_	0.16***	0.48***	0.07**	0.23***	−0.31***	0.25***	–			
	Extr_2_	0.09***	0.07**	0.47***	0.10***	−0.21***	0.33***	0.24***	–		
	Agr_2_	0.09***	0.21***	0.09***	0.44***	−0.27***	0.20***	0.32***	0.15***	–	
	Neu_2_	−0.14***	−0.29***	-0−22***	−0.29***	0.46***	−0.23***	−0.47***	−0.41***	−0.44***	–

The personality traits are Openness (Open), Conscientiousness (Cons), Extroversion (Extr), Agreeableness (Agr), and Neuroticism (Neu).

***p < 0.001, correlation is significant at the 0.01 level (2-tailed).

**p < 0.01, correlation is significant at the 0.05 level (2-tailed).

*N* = 3,411. The bold values represent the correlation of measuring the same traits using different methods.

### 3.3. Comparation between the full model and the LIWC model

The Pearson correlation coefficients between test scores and predicted scores of the “full model” and the “LIWC model” are listed in the Table 3. The results show that the “full model” performed better than the “LIWC model” in cross-validation.

### 3.4. Linguistic feature importance

**FIGURE 4:**
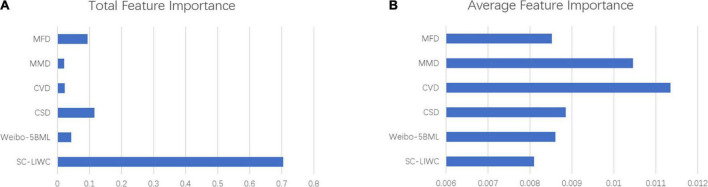
Feature importance of different lexicons. **(A)** The total feature importance of different lexicons. **(B)** The average feature importance of different lexicons.

We found that all 120 linguistic features were used to train the prediction model. As shown in Figure 4, the results of this study showed that the SC-LIWC made the greatest contribution to predicting personality, with a total importance of 70%. However, concerning average feature importance, the word-average importance of the SC-LIWC lexicon was the lowest. The linguistic features of CVD performed the best during the process of model development, followed by MMD, CSD, MFD, and Weibo-5BML. The levels of average importance of the features of these five lexicons were all higher than those of SC-LIWC.

### 3.5. Structural validity

Table 4 presents the zero-order correlation matrix among variables. The numbers along the diagonal line (written in bold) represent the correlations among different methods of measuring the same trait; the numbers in the blue and orange triangles represent the correlations among different traits measured using the same method, and the numbers in the green area represent the correlations among different methods of measuring different traits.

The results showed that the numbers written in bold were all significantly larger than the data contained in the green area in the same column (i.e., the correlation coefficient of the same dimension of different methods was greater than the correlation coefficient of different methods of different dimensions), indicating that our model had good convergent validity. In addition, the bold numbers were all greater than the corresponding values in the orange triangle (i.e., the correlation coefficient of different methods in the same dimension was greater than the correlation coefficient of the same method in different dimensions), with the exception of the dimensions of agreeableness. The results indicated that the discriminant validity of our model was also good.

### 3.6. Criterion validity

The criterion validity of our model is shown in Table 5 (see *R*_1_). When measuring a psychological variable using different assessment instruments or methods, the correlation coefficients between different instruments or methods typically range from approximately 0.39 to 0.68 (60). Therefore, our results showed that the correlation coefficients reached the level of significance, indicating that the models we developed exhibited good criterion validity.

**TABLE 5:** Criterion validity and split-half reliability of each dimension.

Dimension	*R*_1_ (*N* = 3,411)	*R*_2_ (*N* = 682)
Open.	0.46***	0.85***
Cons.	0.48***	0.88***
Extr.	0.47***	0.88***
Agr.	0.44***	0.85***
Neu.	0.46***	0.84***

*R*_1_, the Pearson correlation coefficients between the predicted values of all data and the actual score of the dimension scale. *R*_2_, the Pearson correlation coefficients between the predicted values of the odd “split-half” data and the predicted values of the even “split-half” data.

***p < 0.001.

### 3.7. Split-half reliability

The split-half reliability of the linguistic prediction model is shown in Table 5 (see *R*_2_). All measures reached the level of significance.

## 4. Discussion

The present study introduced domain knowledge to improve the interpretability of a personality prediction model based on social media users’ language habits. We evaluated participants’ personality traits by using five dimensions of the BFI-44 as the output, extracted linguistic features using six lexicons as the input, and ultimately developed a linguistic prediction model for personality recognition. Finally, we tested the validity and reliability of the model by calculating its criterion validity, structural validity, and split-half reliability. The results indicated that the proposed linguistic prediction model has good split-half reliability, criterion validity, and structural validity. However, the discriminant validity of agreeableness was not sufficiently high in this study.

A closed-vocabulary approach was used in this study to introduce domain knowledge to the prediction model. By using this method, we can obtain a clear idea of the features that are most effective in predicting personality. Moreover, although closed-vocabulary analysis was proven not to be as effective as open-vocabulary analysis (32), our model nevertheless achieved relatively advanced prediction accuracy (13). This result also proves the importance of introducing domain knowledge training prediction models from the side. Another piece of evidence regarding the importance of this strategy lies in the fact that our results show that the more closely related variables are to personality, the more effective their corresponding features are in the context of model training. A detailed discussion of these results is as follows.

First, our results showed that SC-LIWC features made the highest overall contribution to predicting personality in this study, achieving a total importance of 70%. This finding is consistent with the results of numerous previous studies that used LIWC as a closed-vocabulary method of extracting social media features (33, 61, 62). The finding also provides evidence to support the validity of SC-LIWC (31). However, regarding the average importance of these six lexicons, that of the SC-LIWC lexicon was the lowest. Besides, the cross-validation result also showed that the prediction accuracy of the “LIWC model” is lower than that of the “full model.” Based on previous studies, we speculate that this deficiency is due to the fact that SC-LIWC is a general psycholinguistic lexicon. We were able to use SC-LIWC to distinguish among people’s emotional states, intentions, thinking styles, and individual differences, but SC-LIWC is not a targeted measurement of personality or other psychological traits that relate to personality. This characteristic may be why the linguistic features extracted using the other five lexicons based on domain knowledge exhibited better performance (18, 61).

We also found that CVD features exhibited the best performance with respect to training the personality prediction model. A large body of literature has suggested that personality is shaped by both genetic and environmental influences. Among the most important of the latter group are cultural influences (63). Culture includes patterns of socialization that shape personality (64). For example, Grimm et al. provided proof that differences exist between collectivists and individualists in terms of their self-described personality traits (65). Our results are consistent with the conclusions of previous studies and provide new evidence for the study of culture and personality.

Furthermore, the linguistic features of MMD and MFD performed better than SC-LIWC features but were far less effective than CVD features. We speculate that this difference may be due to the fact that the relationship between morality and personality remains controversial and is not as stable as the relationship between culture and personality. The field of moral development includes two opposing views regarding the existence of a connection between morality and personality. According to one way of discussing moral development, the stronger connection is between moral agency and personality (66). However, two obstacles remain in this domain. First, precise descriptions of the developmental or influencing processes that are operative in the relationship between morality and personality remain lacking. Second, due to the complex ways in which personality is understood, it remains unclear which of the various options for conceptualizing personality is the best candidate for a developmental analysis of the moral field (66). The findings of this study indirectly verify the connection between morality and personality and provide evidence for research concerning moral development. In the future, more in-depth research can be conducted to investigate the two defects mentioned previously.

Our findings also suggested that the features extracted from Weibo-5BML performed better than those extracted from SC-LIWC. Although emotional lexicons are often used for personality prediction (39, 40), the fact that the contribution of these lexicons to the personality prediction model in this study was lower than that of lexicons measuring culture and morals was unexpected. In fact, personality and emotion could be conceived of as nested because both describe cybernetic processes. However, the two terms are not synonymous. One previous review indicated that the empirical associations between personality traits and emotion regulation are meaningful but modest in magnitude (38). These facts may explain our results regarding emotional lexicons.

Finally, CSD features also had greater importance for model training than SC-LIWC features. A great deal of literature has reported that patients with borderline personality disorder commit suicide more often than their counterparts in the general population (67). It has also been claimed that certain personality traits may be useful markers of suicide risk (68). The results of previous studies have suggested a link between specific personalities and suicide risk. Therefore, personality may have some degree of predictive power regarding suicide risk, but not vice versa.

This research not only enhances the interpretability of the machine learning model but also ensures the validity and stability of the model. We draw on the method developed by Wang et al. (58) to measure the multidimensional prediction model’s structural validity, criterion validity, and split-half reliability. Our results show that the prediction model exhibited fairly good structural validity, criterion validity, and split-half reliability. And the personality prediction model outperformed previous model based on similar database (69). However, the discriminant validity result for the dimensions of agreeableness was less than satisfactory. These findings are understandable because people may want to exhibit their good side online, thus making it difficult to distinguish among linguistic expressions. Poor performance with respect to this personality trait was also reported by other studies (6, 70).

We also found that the correlation between the predictive scores of agreeableness and conscientiousness was high. According to one previous study of personality analysis based on social media, a high positive correlation between the agreeableness and conscientiousness of social media users was found by social media text analysis (71). In addition, Gu et al. found that users who were willing to share their personal information (such as their educational information or location) on social media exhibited higher levels of agreeableness and conscientiousness. Therefore, we speculate that due to the active social media user group, those who are willing to share their life on the internet have high levels of conscientiousness and agreeableness, and these two factors are highly positively correlated. This correlation may be the reason for the poor performance of our model with respect to distinguishing between agreeableness and conscientiousness. Future work regarding personality prediction should focus on improving the discriminant validity between different traits.

In summary, this study has positive implications regarding the introduction of machine learning approaches into the field of psychology and psychiatry. First, the reliability and validity of the prediction model were tested using psychological questionnaire preparation methods. The test result exhibited fairly good structural validity, criterion validity, and split-half reliability. Second, this personality prediction model has good interpretability than other previous works. We used many psychological linguistic features to build prediction model and analyzed the feature importance and found relationships between personality and morality, suicide, cultural values, and emotion, respectively, based on this data-driven results. Our study also has two applications on mental health. First, the great performance on reliability and validity of the prediction model offers a solid basis to conduct large-scale user study. Combined with other study, this model can be used as an auxiliary means to help with mental health diagnosis. Second, the data-driven result showed that personality is related to suicide ideation and emotion, it can help us understand the mental health and high-risk factors associated with internet users.

This study also faces some limitations. First, although improving the accuracy of personality prediction was not the purpose of this study, it did not use the extant advanced methods of natural language processing (NLP) to develop the personality prediction model. We chose to improve the interpretability of the statistical prediction model and then to ensure the accuracy of the prediction model. Future research could focus on methods that combine domain knowledge with deep learning methods, such as the use of knowledge graphs, to further improve the accuracy of personality prediction. Second, this study used only six measurement lexicons of psychological traits that may be relevant to personality. A wider range of psychological knowledge could be introduced in the future to make predictions regarding dependent variables. As discussed in this article, this approach could not only improve the interpretability of the statistical prediction model but also determine whether a psychological trait corresponding to the dictionary is related to the dependent variable to some extent. Finally, considering the fact that the participants were randomly recruited on social media, most of the participants in this experiment were female. The sampling was thus biased, although the study tried hard to ensure that the labels were distributed as uniformly as possible. This problem has also affected many previous studies (58, 61, 72), and we must find ways of balancing the gender ratio of the participants in social media research in the future.

## 5. Conclusion

This study developed a multi-objective model by introducing domain knowledge to predict personality based on social media expression. On the basis of ensuring the reliability and validity of the prediction model, we aimed to improve the interpretability of machine learning models. Our research also has positive implications regarding the combination of machine learning approaches and domain knowledge in the field of psychiatry and its applications to mental health.

## Data availability statement

The data sets generated for this article are not readily available because the raw data cannot be made public; if necessary, feature data can be provided. Requests to access the data sets should be directed to the corresponding author.

## Ethics statement

The Ethical Conduct of the research was approved by the Institutional Review Board at the Institute of Psychology, Chinese Academy of Sciences, under code H15009. The patients/participants provided their written informed consent to participate in this study.

## Author contributions

NH, XL, and TZ conceived and planned this manuscript. NH, SL, and FH carried out the search and revision of the literature. TZ collected and provided the data. NH, YS, and YW analyzed the data. NH drafted the study. XL, LL, and TZ reviewed and edited the writing. All authors revised the manuscript critically for important intellectual content, commented on and approved the final manuscript, were accountable for all aspects of the work, read, and agreed to the published version of the manuscript.

## Footnotes

1
http://www.ocf.berkeley.edu/~johnlab/bfi.htm
